# Comparative post-marketing reporting signals of elagolix and myfembree in endometriosis: a FAERS pharmacovigilance study

**DOI:** 10.3389/fphar.2026.1860816

**Published:** 2026-06-05

**Authors:** MengLong Bai, Shiju Shen, Jin-Yuan Chen

**Affiliations:** 1 Department of Pharmacy, Women and Children’s Hospital, School of Medicine, Xiamen University, Xiamen, China; 2 Charite - Universitatsmedizin Berlin, Berlin, Germany; 3 Central Laboratory, The First Affiliated Hospital, Fujian Medical University, Fuzhou, China

**Keywords:** disproportionality analysis, elagolix, endometriosis, FAERS, myfembree, pharmacovigilance

## Abstract

**Background:**

Elagolix and Myfembree are gonadotropin-releasing hormone (GnRH)-pathway therapies used for endometriosis, but their post-marketing safety reporting patterns remain incompletely characterized. Because spontaneous reporting databases are susceptible to reporting bias and differential market exposure, comparative analyses require cautious interpretation.

**Methods:**

We analyzed quarterly data from the FDA Adverse Event Reporting System (FAERS) from 2015Q3 to 2026Q1. Deduplicated female reports with endometriosis-related indications were identified and classified as elagolix, Myfembree, or other endometriosis-related reports. The primary analysis consisted of drug-specific case-noncase disproportionality analyses for elagolix and Myfembree separately within the female endometriosis reporting background. A direct elagolix-versus-Myfembree head-to-head analysis was performed as a secondary analysis. Reporting odds ratios (RORs), proportional reporting ratios (PRRs), and information components (ICs) were calculated at the preferred term (PT) level. Sensitivity analyses included serious-report-only, healthcare-professional-only, physician-only, complete-age, reporter-type-stratified, overlapping-market-period, and bootstrap analyses.

**Results:**

A total of 4,428 deduplicated female endometriosis-related reports were included, comprising 1,744 elagolix reports, 280 Myfembree reports, and 2,404 other endometriosis-related reports. Serious reports accounted for 31.2% of elagolix reports and 26.8% of Myfembree reports. In drug-specific case-noncase analyses, elagolix showed robust disproportionality signals for hot flush, night sweats, and suicidal ideation. Myfembree showed distinct reporting signals for reproductive and bleeding-related PTs, including heavy menstrual bleeding and intermenstrual bleeding. In the secondary head-to-head analysis, selected PTs including hot flush, nausea, headache, depression, arthralgia, and suicidal ideation showed higher reporting signals for elagolix, whereas alopecia showed a lower reporting signal for elagolix. Sensitivity analyses using alternative algorithms, reporter-type restrictions, overlapping-market-period restriction, complete-age restriction, and bootstrap validation generally supported the direction of the main selected reporting patterns, although some estimates were limited by small cell counts.

**Conclusions:**

Elagolix and Myfembree showed distinct post-marketing reporting signal profiles among female endometriosis-related FAERS reports. Elagolix was characterized mainly by vasomotor and selected neuropsychiatric reporting signals, whereas Myfembree was characterized mainly by reproductive and bleeding-related reporting signals. These findings represent hypothesis-generating reporting differences rather than clinical incidence rates or causal risk estimates. Further pharmacoepidemiologic studies with denominator data and adjustment for patient-level confounding are needed to clarify comparative safety profiles.

## Background

Endometriosis is a chronic estrogen-dependent gynecologic disorder characterized by the presence of endometrial-like tissue outside the uterine cavity (As-Sanie et al., 2025). It commonly manifests with dysmenorrhea, chronic pelvic pain, dyspareunia, and infertility, and imposes a substantial burden on physical, psychological, and reproductive health (Zondervan et al., 2020; Della Corte et al., 2020). Because the disease often affects women during their reproductive years and tends to recur, long-term medical management is frequently required (Becker et al., 2022).

Hormonal suppression remains a cornerstone of endometriosis treatment. In recent years, gonadotropin-releasing hormone (GnRH) antagonists have emerged as important therapeutic options because they suppress ovarian hormone production and alleviate endometriosis-associated symptoms (Taylor et al., 2017). Elagolix, an oral GnRH receptor antagonist, has been widely used for pain control in women with endometriosis (Leyland et al., 2021). However, by inducing a hypoestrogenic state, elagolix may also be associated with adverse events such as vasomotor symptoms, mood disturbances, sleep disorders, and musculoskeletal complaints (Surrey et al., 2018). These safety concerns are clinically relevant, particularly when treatment is prolonged or when patients have preexisting psychiatric vulnerability (Leyland et al., 2021; Surrey et al., 2018).

Myfembree, a fixed-dose combination of relugolix, estradiol, and norethindrone acetate, represents a related but pharmacologically distinct strategy (Giudice et al., 2022). In contrast to elagolix monotherapy, Myfembree incorporates add-back therapy, which is intended to reduce the consequences of estrogen suppression while maintaining therapeutic efficacy (Becker et al., 2024). This difference in formulation may influence post-marketing reporting patterns, especially for symptoms related to hypoestrogenic effects and reproductive-system events (Syed, 2022; Blair, 2024).

Although both therapies are increasingly used in clinical practice, direct real-world comparisons of their safety profiles remain limited. Clinical trials provide important evidence regarding efficacy and common adverse events, but they may not fully capture uncommon, delayed, or heterogeneous events observed after marketing. As a large spontaneous reporting system, the FDA Adverse Event Reporting System (FAERS) provides an opportunity to evaluate post-marketing adverse event patterns in a broader patient population.

Therefore, the present study aimed to characterize drug-specific and comparative post-marketing reporting signal profiles for elagolix and Myfembree among female endometriosis-related FAERS reports. By applying drug-specific case-noncase disproportionality analyses, secondary head-to-head comparisons, and multiple sensitivity analyses, we sought to identify clinically relevant reporting patterns and provide hypothesis-generating evidence to inform future pharmacoepidemiologic research and post-marketing safety monitoring.

## Methods

### Data source

Data were obtained from the FDA Adverse Event Reporting System (FAERS), a publicly available spontaneous reporting database that collects adverse event reports submitted by healthcare professionals, manufacturers, and consumers. Quarterly FAERS ASCII files from 2015Q3 to 2026Q1 were downloaded and processed. The FAERS relational tables, including demographic information, drug information, adverse reactions, indications, outcomes, and report sources, were imported and merged using unique report identifiers.

Duplicate reports were handled according to standard FAERS procedures. When multiple versions of the same case were present, the report with the highest case version was retained. If case-version information was unavailable, duplicate records were resolved using the most recent or largest available primary report identifier. All analyses were conducted at the report level, with each preferred term counted once per report.

### Study design and cohort selection

**FIGURE 1:**
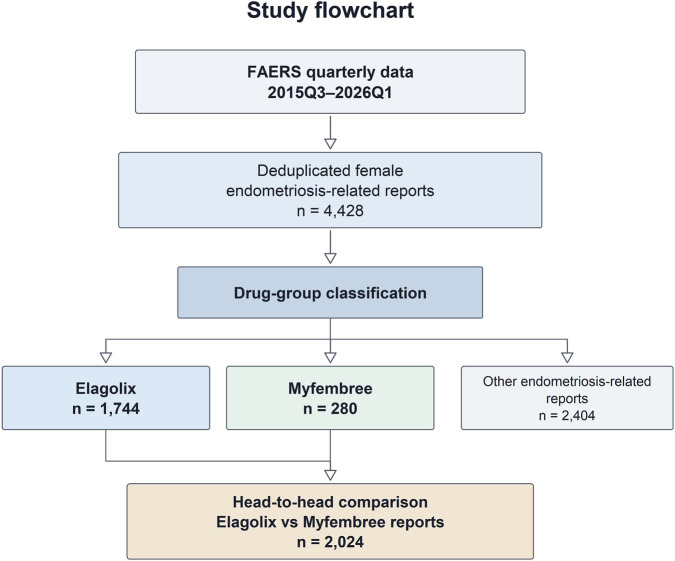
Study flowchart. Flowchart showing the selection and classification of FAERS reports included in this pharmacovigilance study. Quarterly FAERS data from 2015Q3 to 2026Q1 were screened to identify 4,428 deduplicated female endometriosis-related reports. These reports were classified into elagolix reports (n = 1,744), Myfembree reports (n = 280), and other endometriosis-related reports (n = 2,404). The direct head-to-head comparison included elagolix and Myfembree reports only, with a total of 2,024 reports.

This retrospective pharmacovigilance study was designed to characterize post-marketing reporting signal profiles for elagolix and Myfembree among female endometriosis-related FAERS reports. The cohort selection workflow is shown in Figure 1.

Reports were eligible if they met the following criteria: female sex, an indication term related to endometriosis, and sufficient drug information to classify the report into an exposure group. Endometriosis-related reports were identified from indication terms including “endometriosis,” “endometriosis pain,” “endometriosis-associated pain,” and related variants. Drug exposure was determined from drug names and active ingredient fields. Elagolix reports were identified using generic and brand names, including “elagolix” and “Orilissa.” Myfembree reports were identified using “Myfembree” and component-based terms related to relugolix, estradiol, and norethindrone/norethisterone combinations.

For the primary drug classification, reports were categorized as elagolix, Myfembree, or other endometriosis-related reports. The primary exposure definition used reports in which the drug was recorded as a primary suspect or secondary suspect drug. Reports containing both elagolix and Myfembree were flagged separately and excluded from direct head-to-head comparisons. The final analysis included 4,428 deduplicated female endometriosis-related reports, including 1,744 elagolix reports, 280 Myfembree reports, and 2,404 other endometriosis-related reports. The direct head-to-head comparison included 2,024 reports involving elagolix or Myfembree.

### Definition and classification of adverse events

Adverse events were analyzed at the preferred term level according to the Medical Dictionary for Regulatory Activities terminology available in FAERS. Preferred terms were used for the primary signal-detection analyses because they provide clinically granular information on specific reported events. System organ class analyses were used descriptively to summarize broader reporting patterns.

Serious reports were identified using FAERS outcome codes. A report was classified as serious if it contained at least one of the following outcome codes: death, life-threatening event, hospitalization, disability, congenital anomaly, required intervention to prevent impairment or damage, or other serious medically important outcome. Reports without these outcome codes were classified as non-serious.

Reporter type was categorized based on available occupation or report-source information. Reports were grouped as healthcare-professional reports, consumer reports, or other/unknown reports. Physician-submitted reports were additionally identified when physician-specific reporting information was available.

Age information was extracted from the demographic table when available. Because FAERS contains age in different units, age was converted to years when possible. Reports with age units recorded as years were used directly, months were divided by 12, weeks by 52.25, days by 365.25, and decades were multiplied by 10. Age availability and missingness were summarized transparently in the baseline table.

### Primary signal-detection analysis

The primary analysis consisted of drug-specific case-noncase disproportionality analyses performed separately for elagolix and Myfembree within the female endometriosis-related reporting background. For elagolix, reports involving elagolix were compared with all non-elagolix female endometriosis-related reports, including Myfembree and other endometriosis-related reports. For Myfembree, reports involving Myfembree were compared with all non-Myfembree female endometriosis-related reports, including elagolix and other endometriosis-related reports.

For each preferred term, a 2 × 2 contingency table was constructed. In the drug-specific case-noncase analysis, a represented the number of reports with the target drug and target preferred term, b represented the number of reports with the target drug but without the target preferred term, c represented the number of comparator reports with the target preferred term, and d represented the number of comparator reports without the target preferred term.

The reporting odds ratio was calculated as:

$$\text{ROR}=\left(a/b\right)/\left(c/d\right)=\left(a\times d\right)/\left(b\times c\right)$$

In addition to ROR, proportional reporting ratio and information component were calculated as complementary disproportionality metrics. The proportional reporting ratio was calculated as:

$$\text{PRR}=\left[a/\left(a+b\right)\right]/\left[c/\left(c+d\right)\right]$$

The information component was calculated using an approximate Bayesian framework:

$$\text{IC}=\log2\left[\left(a+0.5\right)/\left(E+0.5\right)\right]$$

where:

$$E=\left[\left(a+b\right)\left(a+c\right)\right]/\left(a+b+c+d\right)$$

The lower 95% credibility interval for IC was expressed as IC025. When any cell in the 2 × 2 table was zero, a Haldane-Anscombe correction was applied by adding 0.5 to all four cells.

Signals were classified using predefined criteria. A basic signal was defined as at least three target preferred-term reports and a lower 95% confidence interval for ROR greater than 1. A robust signal was defined as at least five target preferred-term reports, a lower 95% confidence interval for ROR greater than 1, PRR ≥ 2, and IC025 > 0. A strict signal was defined as at least ten target preferred-term reports, a lower 95% confidence interval for ROR greater than 1, PRR ≥ 2, and IC025 > 0. Estimates based on small cell counts or zero-cell corrections were flagged as potentially unstable.

### Secondary head-to-head analysis

A direct elagolix-versus-Myfembree head-to-head analysis was performed as a secondary analysis. This analysis was restricted to reports classified as elagolix or Myfembree and excluded other endometriosis-related reports and reports containing both drugs. For each preferred term, elagolix reports were compared directly with Myfembree reports using the same ROR, PRR, IC, and IC025 framework described above.

Because of the substantial difference in the number of reports between elagolix and Myfembree, the head-to-head analysis was interpreted as a secondary comparative signal analysis rather than as the sole basis for inference. Clinically selected preferred terms were visualized in forest plots, while complete PT-level results were provided in supplementary tables.

### Sensitivity, stratified, and robustness analyses

Several sensitivity and stratified analyses were performed to evaluate the robustness of the reporting patterns and to address potential reporting bias.

First, analyses were repeated after restricting the dataset to serious reports only. Second, analyses were repeated among healthcare-professional-submitted reports to reduce the influence of consumer reporting. Third, a physician-only sensitivity analysis was performed when physician-specific reporter information was available. Fourth, reporter-type-stratified analyses were conducted separately for consumer and healthcare-professional reports.

To address differential market tenure and the potential Weber effect, an overlapping-market-period analysis was conducted by restricting reports to the period after Myfembree became available for endometriosis-related use. Annual reporting trends were also summarized by calendar year for elagolix and Myfembree. Data for 2026 were considered partial-year data because only Q1 reports were included.

To evaluate the influence of age missingness, an exploratory complete-age sensitivity analysis was conducted among reports with complete or convertible age information. This analysis was interpreted cautiously because age was missing in a substantial proportion of FAERS reports. Multiple imputation was not performed because FAERS lacks sufficient patient-level covariates to support reliable imputation and because the missingness mechanism could not be assumed to be random.

Bootstrap validation was performed for selected preferred terms to assess the directional stability of key head-to-head estimates. Bootstrap resampling was conducted at the report level, and the proportion of bootstrap estimates with ROR greater than one was summarized for each selected preferred term. Bootstrap results were used as supportive robustness information and were not interpreted as evidence of causality.

Top PT-level signals for each drug were also tabulated to ensure that results were not limited to selected clinically highlighted terms.

### Data visualization

All analyses and visualizations were performed using R software. Flow diagrams were used to summarize cohort selection. Forest plots were generated to display drug-specific case-noncase reporting signals and selected head-to-head reporting signals. Additional supplementary figures were used to show overlapping-market-period analyses, annual reporting trends, serious-reporting associations, and the distribution of selected preferred terms by serious reporting status. All visualizations were interpreted as descriptive or disproportionality-based reporting patterns rather than incidence-based safety estimates.

### Ethical considerations

This study used publicly available, de-identified FAERS data and involved no direct patient contact or intervention. Therefore, institutional review board approval and informed consent were not required.

## Results

### Study population and data selection

A total of 4,428 deduplicated female endometriosis-related reports were identified from FAERS quarterly data between 2015Q3 and 2026Q1. After drug-group classification, 1,744 reports were assigned to elagolix, 280 reports to Myfembree, and 2,404 reports to other endometriosis-related reports. The direct head-to-head comparison between elagolix and Myfembree included 2,024 reports. The study flowchart is shown in Figure 1.

The baseline characteristics of elagolix and Myfembree reports are summarized in Table 1. Serious reports accounted for 31.2% of elagolix reports and 26.8% of Myfembree reports. Age information was incomplete in both groups, but missing age information was more common among elagolix reports than Myfembree reports (62.2% vs. 43.2%). Among reports with available age data, the median age was 31 years for elagolix and 34 years for Myfembree. The reporting periods also differed between the two drugs, with elagolix reports spanning 2018–2026 and Myfembree reports spanning 2022–2026. Regarding reporter type, healthcare-professional reports accounted for 60.3% of elagolix reports and 78.9% of Myfembree reports, whereas consumer reports accounted for 31.1% and 11.1%, respectively.

**TABLE 1:** Baseline characteristics of elagolix and Myfembree reports.

Characteristic	Elagolix	Myfembree
Reports, n	1,744	280
Serious reports, n (%)	545 (31.2%)	75 (26.8%)
Age available, n (%)	660 (37.8%)	159 (56.8%)
Age missing/unavailable, n (%)	1,084 (62.2%)	121 (43.2%)
Age, median (IQR), years	31 (25–37)	34 (25.8–40)
Report years	2018–2026	2022–2026
Consumer reports, n (%)	543 (31.1%)	31 (11.1%)
Healthcare-professional reports, n (%)	1,052 (60.3%)	221 (78.9%)
Other/unknown reports, n (%)	149 (8.5%)	28 (10.0%)
Physician reports, n	534	65
Pharmacist reports, n	26	4
Health-professional reports, n	81	15
Other health-professional reports, n	66	0

Values are presented as n, n (%), or median (IQR), as appropriate. HCP, healthcare professional; IQR, interquartile range.

### Drug-specific case-noncase reporting signals

**FIGURE 2:**
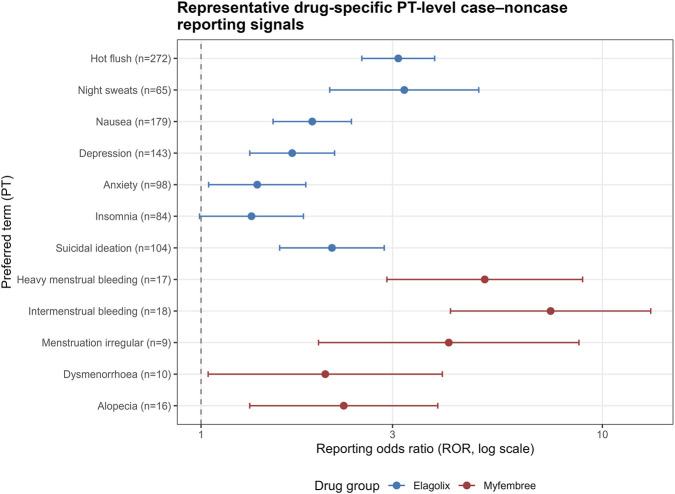
Representative drug-specific PT-level case-noncase reporting signals. Forest plot showing representative drug-specific preferred term (PT)-level case-noncase reporting signals for elagolix and Myfembree within the female endometriosis-related FAERS reporting background. Blue points represent elagolix-related signals, and red points represent Myfembree-related signals. Points indicate reporting odds ratios (RORs), and horizontal bars indicate 95% confidence intervals. The vertical dashed line indicates ROR = 1. Elagolix showed representative reporting signals for hot flush, night sweats, nausea, depression, anxiety, insomnia, and suicidal ideation, whereas Myfembree showed representative reporting signals for heavy menstrual bleeding, intermenstrual bleeding, menstruation irregular, dysmenorrhoea, and alopecia.

Drug-specific case-noncase analyses were performed separately for elagolix and Myfembree within the female endometriosis-related reporting background. Representative PT-level results are shown in Table 2; Figure 2.

**TABLE 2:** Representative drug-specific case-noncase reporting signals.

Drug	Preferred term	Cases, n	ROR (95% CI)	PRR (95% CI)	IC/ IC025	Signal classification	Clinical category
Elagolix	Hot flush	272	3.10 (2.52–3.82)	2.77 (2.29–3.35)	0.71/0.43	Strict/ robust signal	Vasomotor/ hypoestrogenic
Elagolix	Night sweats	65	3.21 (2.09–4.92)	3.13 (2.06–4.75)	0.76/0.19	Strict/ robust signal	Vasomotor/ hypoestrogenic
Elagolix	Suicidal ideation	104	2.12 (1.57–2.86)	2.05 (1.54–2.74)	0.53/0.10	Strict/ robust signal	Neuropsychiatric/ sleep-related
Elagolix	Depression	143	1.69 (1.32–2.15)	1.63 (1.30–2.05)	0.38/0.03	Basic signal	Neuropsychiatric/ sleep-related
Elagolix	Anxiety	98	1.38 (1.04–1.82)	1.36 (1.04–1.77)	0.25/-0.17	Basic signal	Neuropsychiatric/ sleep-related
Elagolix	Insomnia	84	1.34 (0.99–1.80)	1.32 (0.99–1.75)	0.23/-0.23	No robust signal	Neuropsychiatric/ sleep-related
Elagolix	Mood swings	68	1.56 (1.11–2.20)	1.54 (1.11–2.14)	0.34/-0.17	Basic signal	Neuropsychiatric/ sleep-related
Elagolix	Arthralgia	125	1.58 (1.22–2.04)	1.54 (1.21–1.96)	0.34/-0.04	Basic signal	Musculoskeletal
Elagolix	Nausea	179	1.89 (1.51–2.37)	1.80 (1.46–2.22)	0.45/0.13	Basic signal	Gastrointestinal
Elagolix	Headache	176	1.51 (1.22–1.87)	1.46 (1.20–1.77)	0.30/-0.02	Basic signal	Other selected PTs
Myfembree	Heavy menstrual bleeding	17	5.09 (2.90–8.93)	4.84 (2.84–8.26)	1.85/0.40	Strict/ robust signal	Reproductive/ bleeding-related
Myfembree	Intermenstrual bleeding	18	7.43 (4.18–13.20)	7.02 (4.06–12.13)	2.19/0.64	Strict/ robust signal	Reproductive/ bleeding-related
Myfembree	Dysmenorrhoea	10	2.04 (1.04–3.99)	2.00 (1.05–3.83)	0.85/-0.61	Basic signal	Reproductive/ bleeding-related
Myfembree	Menstruation irregular	9	4.14 (1.96–8.74)	4.04 (1.95–8.36)	1.59/-0.25	Basic signal	Reproductive/ bleeding-related
Myfembree	Alopecia	16	2.27 (1.32–3.89)	2.19 (1.32–3.66)	0.98/-0.22	Basic signal	Other selected PTs

Drug-specific case-noncase analyses compared reports involving the target drug with all non-target-drug reports within the female endometriosis-related FAERS, reporting background. PTs, shown are representative clinically relevant reporting signals selected for main-text presentation. ROR, reporting odds ratio; PRR, proportional reporting ratio; IC, information component; IC025, lower 95% credibility interval of the information component; CI, confidence interval.

For elagolix, robust and strict disproportionality signals were observed for vasomotor/hypoestrogenic symptoms, including hot flush (n = 272; ROR = 3.10, 95% CI: 2.52–3.82; PRR = 2.77, 95% CI: 2.29–3.35; IC/IC025 = 0.71/0.43) and night sweats (n = 65; ROR = 3.21, 95% CI: 2.09–4.92; PRR = 3.13, 95% CI: 2.06–4.75; IC/IC025 = 0.76/0.19). A robust and strict signal was also observed for suicidal ideation (n = 104; ROR = 2.12, 95% CI: 1.57–2.86; PRR = 2.05, 95% CI: 1.54–2.74; IC/IC025 = 0.53/0.10). Additional basic signals were observed for depression, anxiety, mood swings, arthralgia, nausea, and headache, although these did not meet the predefined robust-signal criteria. Insomnia showed a directionally elevated but non-significant reporting pattern.

For Myfembree, the most prominent drug-specific signals were reproductive and bleeding-related PTs. Robust and strict signals were observed for intermenstrual bleeding (n = 18; ROR = 7.43, 95% CI: 4.18–13.20; PRR = 7.02, 95% CI: 4.06–12.13; IC/IC025 = 2.19/0.64) and heavy menstrual bleeding (n = 17; ROR = 5.09, 95% CI: 2.90–8.93; PRR = 4.84, 95% CI: 2.84–8.26; IC/IC025 = 1.85/0.40). Other reproductive or menstrual terms, including dysmenorrhoea, menstruation irregular, and alopecia, were included in the representative visualization but did not uniformly meet robust-signal criteria. These findings suggest distinct drug-specific reporting profiles, with elagolix characterized mainly by vasomotor and selected neuropsychiatric reporting signals, whereas Myfembree was characterized mainly by reproductive and bleeding-related reporting signals.

### Secondary head-to-head PT-level comparison

**FIGURE 3:**
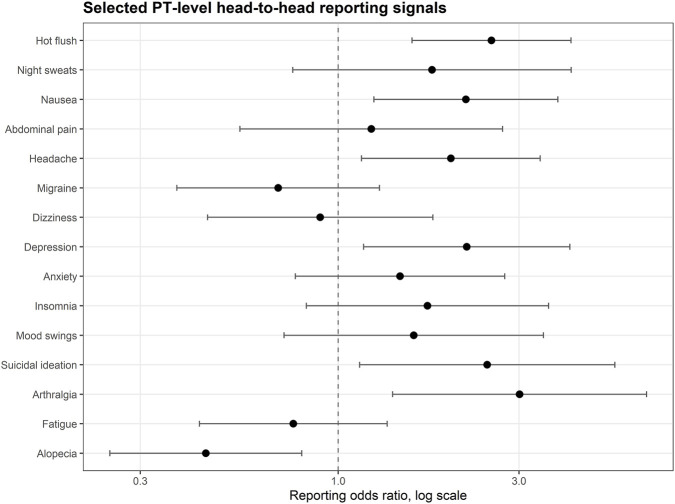
Selected PT-level head-to-head reporting signals. Forest plot showing selected preferred term (PT)-level reporting signals in the secondary head-to-head comparison between elagolix and Myfembree. Points represent reporting odds ratios (RORs), and horizontal bars indicate 95% confidence intervals. The vertical dashed line indicates ROR = 1. Values greater than 1 indicate relatively higher reporting for elagolix, whereas values less than 1 indicate relatively higher reporting for Myfembree. Hot flush, nausea, headache, depression, suicidal ideation, and arthralgia showed higher reporting signals for elagolix, whereas alopecia showed a lower reporting signal for elagolix. These results should be interpreted as comparative reporting patterns rather than clinical incidence or causal risk estimates.

A direct elagolix-versus-Myfembree head-to-head analysis was performed as a secondary comparative signal analysis. Selected clinically relevant PT-level results are summarized in Table 3 and visualized in Figure 3.

**TABLE 3:** Selected PT-level head-to-head reporting signals.

Preferred term	Elagolix reports, n	Myfembree reports, n	ROR (95% CI)	PRR (95% CI)	IC/IC025	Signal classification	Clinical category
Hot flush	272	19	2.54 (1.57–4.12)	2.30 (1.47–3.60)	0.12/-0.13	Basic signal	Vasomotor/ hypoestrogenic
Night sweats	65	6	1.77 (0.76–4.12)	1.74 (0.76–3.98)	0.09/-0.41	No robust signal	Vasomotor/ hypoestrogenic
Nausea	179	14	2.17 (1.24–3.80)	2.05 (1.21–3.48)	0.11/-0.20	Basic signal	Gastrointestinal
Abdominal pain	53	7	1.22 (0.55–2.72)	1.22 (0.56–2.65)	0.04/-0.51	No robust signal	Gastrointestinal
Headache	176	15	1.98 (1.15–3.41)	1.88 (1.13–3.14)	0.10/-0.21	Basic signal	Other selected PTs
Migraine	57	13	0.69 (0.37–1.28)	0.70 (0.39–1.27)	−0.08/-0.60	No robust signal	Other selected PTs
Dizziness	56	10	0.90 (0.45–1.78)	0.90 (0.46–1.74)	−0.02/-0.55	No robust signal	Other selected PTs
Depression	143	11	2.18 (1.17–4.09)	2.09 (1.15–3.80)	0.11/-0.23	Basic signal	Neuropsychiatric/ sleep-related
Anxiety	98	11	1.46 (0.77–2.75)	1.43 (0.78–2.63)	0.06/-0.35	No robust signal	Neuropsychiatric/ sleep-related
Insomnia	84	8	1.72 (0.82–3.59)	1.69 (0.83–3.44)	0.08/-0.36	No robust signal	Neuropsychiatric/ sleep-related
Mood swings	68	7	1.58 (0.72–3.48)	1.56 (0.72–3.36)	0.07/-0.42	No robust signal	Neuropsychiatric/ sleep-related
Suicidal ideation	104	7	2.47 (1.14–5.37)	2.39 (1.12–5.07)	0.12/-0.28	Basic signal	Neuropsychiatric/ sleep-related
Arthralgia	125	7	3.01 (1.39–6.52)	2.87 (1.35–6.07)	0.14/-0.23	Basic signal	Musculoskeletal
Fatigue	72	15	0.76 (0.43–1.35)	0.77 (0.45–1.32)	−0.06/-0.52	No robust signal	Other selected PTs
Alopecia	46	16	0.45 (0.25–0.80)	0.46 (0.27–0.80)	−0.21/-0.78	No robust signal	Skin/ subcutaneous

The head-to-head analysis directly compared elagolix and Myfembree reports and was interpreted as a secondary comparative signal analysis. ROR, reporting odds ratio; PRR, proportional reporting ratio; IC, information component; IC025, lower 95% credibility interval of the information component; PT, preferred term; CI, confidence interval.

In the head-to-head comparison, several selected PTs showed higher reporting signals for elagolix than for Myfembree. Basic signals were observed for hot flush (n = 272 vs. 19; ROR = 2.54, 95% CI: 1.57–4.12), nausea (n = 179 vs. 14; ROR = 2.17, 95% CI: 1.24–3.80), headache (n = 176 vs. 15; ROR = 1.98, 95% CI: 1.15–3.41), depression (n = 143 vs. 11; ROR = 2.18, 95% CI: 1.17–4.09), suicidal ideation (n = 104 vs. 7; ROR = 2.47, 95% CI: 1.14–5.37), and arthralgia (n = 125 vs. 7; ROR = 3.01, 95% CI: 1.39–6.52). Night sweats, anxiety, insomnia, and mood swings showed directionally elevated RORs for elagolix but did not meet the basic signal threshold because their confidence intervals crossed the null value. In contrast, alopecia showed a lower reporting signal for elagolix than for Myfembree (n = 46 vs. 16; ROR = 0.45, 95% CI: 0.25–0.80).

Importantly, none of the selected PTs in the head-to-head analysis fulfilled the predefined robust-signal criteria based on concordant ROR, PRR, and IC025 thresholds. Therefore, these findings were interpreted as secondary comparative reporting patterns rather than as evidence of clinical incidence differences.

### Sensitivity, stratified, and robustness analyses

Several sensitivity and stratified analyses were performed to evaluate the robustness of the selected reporting patterns. In the overlapping-market-period analysis, hot flush remained a robust signal (ROR = 3.97, 95% CI: 2.33–6.76), while night sweats (ROR = 2.64, 95% CI: 1.06–6.57), nausea (ROR = 2.68, 95% CI: 1.45–4.94), headache (ROR = 2.88, 95% CI: 1.60–5.20), and arthralgia (ROR = 3.32, 95% CI: 1.45–7.62) showed basic reporting signals. Alopecia continued to show a lower reporting signal for elagolix during the overlapping market period (ROR = 0.24, 95% CI: 0.09–0.62). These results are shown in Supplementary Table S7; Supplementary Figure S1.

Bootstrap validation supported the directional stability of several selected head-to-head estimates, including hot flush, nausea, headache, depression, arthralgia, suicidal ideation, anxiety, insomnia, mood swings, and night sweats. However, bootstrap stability was interpreted as supportive evidence for directional consistency rather than as evidence of causality or incidence-based risk differences. Bootstrap results are provided in Supplementary Table S8.

Exploratory complete-age sensitivity analysis was performed among reports with complete or convertible age information. This analysis included 660 elagolix reports and 159 Myfembree reports with available age data. The directions of selected reporting signals were broadly consistent for several PTs, including hot flush (ROR = 2.26, 95% CI: 1.18–4.33) and nausea (ROR = 2.70, 95% CI: 1.22–5.98), although confidence intervals widened because of the reduced sample size. Complete-age sensitivity results are shown in Supplementary Table S12.

Physician-only sensitivity analysis included 534 elagolix reports and 65 Myfembree reports. Owing to the limited number of Myfembree physician-submitted reports, several estimates were flagged as small-cell or unstable. Nevertheless, the physician-only analyses were included as an additional assessment of reporter-type bias. The full physician-only results are provided in Supplementary Table S13.

Annual reporting trends were also summarized to assess potential differential market tenure and Weber effect. Elagolix reports were most frequent in the earlier post-approval years, whereas Myfembree reports appeared mainly from 2022 onward. During the overlapping reporting period, both drugs contributed reports, supporting the use of overlapping-period sensitivity analysis. Data for 2026 included Q1 only and were not interpreted as full-year trends. Annual reporting trends are shown in Supplementary Table S11; Supplementary Figure S4.

Expanded analyses, including full PT-level head-to-head results, complete drug-specific case-noncase results, serious-only analyses, healthcare-professional-only analyses, reporter-type-stratified analyses, top 20 PT-level signal tables for each drug, and descriptive serious-reporting analyses, are provided in the (Supplementary Tables S1-S4; Supplementary Figures S1-S4). Overall, the supplementary analyses supported the presence of distinct post-marketing reporting profiles for elagolix and Myfembree while reinforcing the need for cautious interpretation of small-cell estimates and spontaneous reporting data.

## Discussion

In this FAERS-based pharmacovigilance study, elagolix and Myfembree showed distinct post-marketing reporting signal profiles among female endometriosis-related reports. In drug-specific case-noncase analyses, elagolix was characterized mainly by vasomotor and selected neuropsychiatric reporting signals, including hot flush, night sweats, and suicidal ideation, whereas Myfembree was characterized mainly by reproductive and bleeding-related reporting signals, including heavy menstrual bleeding and intermenstrual bleeding. These findings may be pharmacologically plausible in the context of differences in GnRH-pathway modulation and hormonal add-back design, but FAERS data cannot distinguish true clinical differences from differences in reporting propensity, baseline patient characteristics, or surveillance patterns.

Neuropsychiatric reporting signals were an important component of the elagolix reporting profile. Elagolix showed selected neuropsychiatric reporting signals, particularly suicidal ideation, and secondary head-to-head analyses also showed elevated reporting signals for depression and suicidal ideation. Because elagolix suppresses ovarian hormone production, the observed vasomotor and neuropsychiatric reporting patterns are pharmacologically plausible (Dorsey et al., 2020), as estrogen withdrawal has been associated with vasomotor symptoms, sleep disturbance, and mood-related symptoms (Haufe et al., 2022; Schmidt et al., 2015; Maki et al., 2026). Myfembree combines relugolix with estradiol and norethindrone acetate as hormonal add-back therapy, a design intended to reduce hypoestrogenic effects during GnRH antagonist therapy (Arjona Ferreira and Migoya, 2023). The lower relative reporting of selected vasomotor and neuropsychiatric PTs for Myfembree in the secondary head-to-head analysis is directionally consistent with this pharmacological rationale; however, FAERS cannot determine whether this pattern reflects a true clinical difference, differential reporting behavior, baseline symptom differences, or differences in drug utilization. Because FAERS disproportionality analyses are intended for signal detection rather than causal inference, these findings should be regarded as hypothesis-generating reporting signals rather than causal or incidence-based safety estimates. Overall, these reporting differences may reflect a combination of pharmacological effects, baseline vulnerability, indication-related symptom burden, reporting propensity, and surveillance patterns.

Elagolix also showed drug-specific reporting signals for hot flush and night sweats, and secondary head-to-head analyses showed higher selected reporting signals for nausea and arthralgia. (Taylor et al., 2017; Dorsey et al., 2020). This pattern is broadly consistent with clinical development data showing dose-dependent hypoestrogenic effects with elagolix (Kingsberg et al., 2023). Relugolix combination therapy was designed to reduce hypoestrogenic effects through hormonal add-back (Arjona Ferreira and Migoya, 2023), but the relatively lower reporting of selected vasomotor PTs for Myfembree in FAERS cannot be attributed to add-back therapy alone. Because vasomotor symptoms, sleep disturbance, and mood symptoms may cluster clinically, these reporting patterns may help prioritize safety monitoring in patients who are potentially vulnerable to hypoestrogenic symptoms (DePree et al., 2023). However, FAERS disproportionality analyses are hypothesis-generating and should not be interpreted as evidence of causality, clinical incidence, or comparative risk (Michel et al., 2017; Scosyrev et al., 2025; Fusaroli et al., 2024).

The Myfembree reporting profile differed from that of elagolix and was characterized mainly by reproductive and bleeding-related PTs. In drug-specific case-noncase analyses, heavy menstrual bleeding and intermenstrual bleeding showed robust disproportionality signals. This pattern may reflect the pharmacological composition of Myfembree, the underlying gynecologic context of endometriosis treatment, or differential reporting of menstrual symptoms. However, spontaneous reporting data cannot distinguish treatment-emergent events from disease-related symptoms, treatment discontinuation effects, background menstrual abnormalities, or differential reporting behavior. Therefore, these reproductive and bleeding-related signals should be interpreted as hypothesis-generating.

A key consideration in comparing elagolix and Myfembree is their difference in market availability, report volume, reporter composition, and data completeness. Elagolix had a longer post-marketing period and more FAERS reports than Myfembree, which may introduce differential surveillance and Weber effect; therefore, we conducted overlapping-market-period and annual trend analyses, in which several selected PT-level reporting patterns remained directionally consistent. We also performed healthcare-professional-only, physician-only, and reporter-type-stratified sensitivity analyses to evaluate the influence of reporting source, although physician-only analyses were limited by the small number of Myfembree reports and should be considered supportive rather than definitive. In addition, age information was frequently missing, which may contribute to residual confounding because age can influence vasomotor, sleep, mood, and gynecologic symptoms. We therefore reported age availability and conducted an exploratory complete-age sensitivity analysis; several selected signals remained directionally consistent, but confidence intervals widened because of reduced sample size. Multiple imputation was not performed because FAERS lacks sufficient patient-level covariates and the missingness mechanism cannot be assumed to be random.

This study has several strengths. First, it used a large, publicly available post-marketing pharmacovigilance database to evaluate reporting patterns for two clinically relevant GnRH-pathway therapies used in endometriosis. Second, rather than relying solely on crude head-to-head RORs, we used drug-specific case-noncase analyses as the primary signal-detection framework and treated direct elagolix-versus-Myfembree comparisons as secondary analyses. Third, multiple disproportionality metrics, including ROR, PRR, and IC/IC025, were calculated, and predefined basic, robust, and strict signal criteria were applied. Fourth, several sensitivity and robustness analyses were performed, including serious-report-only, healthcare-professional-only, physician-only, reporter-type-stratified, overlapping-market-period, complete-age, and bootstrap analyses.

Several limitations should be acknowledged. FAERS is a spontaneous reporting system and is subject to underreporting, stimulated reporting, duplicate reports, missing data, incomplete clinical information, and reporting bias. Disproportionality analyses cannot estimate true incidence, absolute risk, relative risk, or causality. The database does not provide reliable denominators, treatment exposure duration, baseline depression or anxiety status, menopausal status, disease severity, prior treatment history, concomitant medications, or separately prescribed hormonal add-back therapy. Concomitant medications, including antidepressants, analgesics, nonsteroidal anti-inflammatory drugs, and hormonal therapies, may influence both adverse-event occurrence and reporting patterns but could not be fully adjusted for in this analysis. In addition, some PT-level estimates, especially for Myfembree, were based on small numbers of reports and should be interpreted cautiously despite the use of small-cell flags, strict criteria, and bootstrap validation.

## Conclusion

This FAERS pharmacovigilance study identified distinct post-marketing reporting signal profiles for elagolix and Myfembree among female endometriosis-related reports. In drug-specific case-noncase analyses, elagolix was characterized mainly by vasomotor and selected neuropsychiatric reporting signals, including hot flush, night sweats, and suicidal ideation, whereas Myfembree was characterized mainly by reproductive and bleeding-related reporting signals, including heavy menstrual bleeding and intermenstrual bleeding. Secondary head-to-head analyses showed higher selected reporting signals for elagolix for several preferred terms, but these findings should be interpreted cautiously because FAERS cannot provide clinical incidence rates, denominator data, or causal risk estimates. Overall, these results should be regarded as hypothesis-generating post-marketing reporting signals that may help prioritize future pharmacoepidemiologic and prospective studies with appropriate patient-level adjustment.

## Data Availability

The datasets presented in this study can be found in online repositories. The names of the repository/repositories and accession number(s) can be found in the article/Supplementary Material.
