# Supplementary material for: Comparative post-marketing reporting signals of elagolix and myfembree in endometriosis: a FAERS pharmacovigilance study
Source: Front Pharmacol. 2026 Jun 5;17:1860816. doi: 10.3389/fphar.2026.1860816 (PMC13279427; doi:10.3389/fphar.2026.1860816)
Supplement: Supplementary file 3 [file Table1.docx]

**Supplementary Tables**

This document contains supplementary tables suitable for Word-format submission. Full PT-level tables (Tables S1–S3), which contain several hundred rows, are recommended to be submitted separately as an Excel supplementary data file.

## **Table S4. Serious-only sensitivity analysis.**

| **Comparison** | **PT** | **Cases target/comparator** | **ROR (95% CI)** | **PRR (95% CI)** | **IC / IC025** | **Signal classification** | **Clinical category** |
| --- | --- | --- | --- | --- | --- | --- | --- |
| Elagolix vs background | Hot flush | 60/80 | 2.53 (1.79–3.60) | 2.37 (1.72–3.26) | 0.82 / 0.22 | Robust signal | Vasomotor |
| Myfembree vs background | Hot flush | 7/133 | 1.59 (0.72–3.53) | 1.54 (0.74–3.17) | 0.55 / -1.07 | No robust signal | Vasomotor |
| Elagolix vs Myfembree | Hot flush | 60/7 | 1.20 (0.53–2.74) | 1.18 (0.56–2.48) | 0.03 / -0.49 | No robust signal | Vasomotor |
| Elagolix vs background | Nausea | 50/113 | 1.44 (1.01–2.03) | 1.40 (1.01–1.92) | 0.35 / -0.25 | No robust signal | GI |
| Elagolix vs Myfembree | Nausea | 50/5 | 1.41 (0.55–3.67) | 1.38 (0.57–3.34) | 0.05 / -0.52 | No robust signal | GI |
| Myfembree vs background | Nausea | 5/158 | 0.92 (0.37–2.31) | 0.92 (0.39–2.18) | -0.10 / -1.78 | No robust signal | GI |
| Elagolix vs Myfembree | Headache | 38/4 | 1.33 (0.46–3.84) | 1.31 (0.48–3.56) | 0.04 / -0.61 | No robust signal | Other |
| Elagolix vs background | Headache | 38/130 | 0.92 (0.63–1.33) | 0.92 (0.65–1.31) | -0.09 / -0.72 | No robust signal | Other |
| Myfembree vs background | Headache | 4/164 | 0.70 (0.25–1.93) | 0.71 (0.27–1.87) | -0.43 / -2.19 | No robust signal | Other |
| Elagolix vs Myfembree | Depression | 58/3 | 2.86 (0.87–9.36) | 2.66 (0.86–8.28) | 0.11 / -0.42 | No robust signal | Neuropsych/sleep |
| Elagolix vs background | Depression | 58/106 | 1.81 (1.30–2.54) | 1.73 (1.27–2.34) | 0.55 / -0.03 | No robust signal | Neuropsych/sleep |
| Myfembree vs background | Depression | 3/161 | 0.52 (0.16–1.68) | 0.54 (0.18–1.66) | -0.76 / -2.67 | No robust signal | Neuropsych/sleep |
| Elagolix vs Myfembree | Arthralgia | 33/3 | 1.55 (0.46–5.17) | 1.51 (0.48–4.81) | 0.06 / -0.64 | No robust signal | Musculoskeletal |
| Elagolix vs background | Arthralgia | 33/90 | 1.17 (0.77–1.76) | 1.16 (0.79–1.70) | 0.15 / -0.56 | No robust signal | Musculoskeletal |
| Myfembree vs background | Arthralgia | 3/120 | 0.72 (0.22–2.31) | 0.73 (0.24–2.24) | -0.39 / -2.39 | No robust signal | Musculoskeletal |
| Elagolix vs background | Suicidal ideation | 100/78 | 4.73 (3.45–6.47) | 4.04 (3.06–5.35) | 1.21 / 0.70 | Robust signal | Neuropsych/sleep |
| Elagolix vs Myfembree | Suicidal ideation | 100/7 | 2.18 (0.97–4.90) | 1.97 (0.95–4.07) | 0.09 / -0.32 | No robust signal | Neuropsych/sleep |
| Myfembree vs background | Suicidal ideation | 7/171 | 1.21 (0.55–2.69) | 1.19 (0.58–2.45) | 0.23 / -1.29 | No robust signal | Neuropsych/sleep |
| Elagolix vs Myfembree | Anxiety | 32/3 | 1.50 (0.45–5.01) | 1.47 (0.46–4.68) | 0.06 / -0.65 | No robust signal | Neuropsych/sleep |
| Elagolix vs background | Anxiety | 32/91 | 1.12 (0.74–1.69) | 1.11 (0.75–1.64) | 0.11 / -0.61 | No robust signal | Neuropsych/sleep |
| Myfembree vs background | Anxiety | 3/120 | 0.72 (0.22–2.31) | 0.73 (0.24–2.24) | -0.39 / -2.39 | No robust signal | Neuropsych/sleep |
| Myfembree vs background | Insomnia | 3/91 | 0.96 (0.30–3.11) | 0.96 (0.31–2.97) | -0.05 / -2.17 | No robust signal | Neuropsych/sleep |
| Elagolix vs Myfembree | Insomnia | 19/3 | 0.87 (0.25–3.00) | 0.87 (0.26–2.87) | -0.02 / -0.93 | No robust signal | Neuropsych/sleep |
| Elagolix vs background | Insomnia | 19/75 | 0.79 (0.47–1.32) | 0.80 (0.49–1.31) | -0.25 / -1.12 | No robust signal | Neuropsych/sleep |
| Myfembree vs background | Fatigue | 5/137 | 1.07 (0.42–2.69) | 1.07 (0.45–2.52) | 0.08 / -1.65 | No robust signal | Other |
| Elagolix vs Myfembree | Fatigue | 19/5 | 0.51 (0.18–1.40) | 0.52 (0.20–1.36) | -0.15 / -1.03 | No robust signal | Other |
| Elagolix vs background | Fatigue | 19/123 | 0.47 (0.29–0.77) | 0.49 (0.30–0.78) | -0.83 / -1.63 | No robust signal | Other |
| Elagolix vs background | Mood swings | 20/41 | 1.56 (0.91–2.68) | 1.54 (0.91–2.60) | 0.43 / -0.52 | No robust signal | Neuropsych/sleep |
| Elagolix vs Myfembree | Mood swings | 20/2 | 1.39 (0.32–6.07) | 1.38 (0.33–5.77) | 0.05 / -0.84 | No robust signal | Neuropsych/sleep |
| Myfembree vs background | Mood swings | 2/59 | 0.99 (0.24–4.13) | 0.99 (0.25–3.97) | -0.01 / -2.54 | No robust signal | Neuropsych/sleep |
| Myfembree vs background | Night sweats | 4/32 | 3.80 (1.31–11.03) | 3.65 (1.32–10.05) | 1.41 / -1.14 | No robust signal | Vasomotor |
| Elagolix vs background | Night sweats | 15/21 | 2.29 (1.17–4.47) | 2.25 (1.17–4.34) | 0.76 / -0.42 | No robust signal | Vasomotor |
| Elagolix vs Myfembree | Night sweats | 15/4 | 0.50 (0.16–1.56) | 0.52 (0.18–1.51) | -0.15 / -1.14 | No robust signal | Vasomotor |
| Myfembree vs background | Migraine | 7/82 | 2.65 (1.18–5.94) | 2.49 (1.19–5.20) | 1.12 / -0.72 | No robust signal | Other |
| Elagolix vs background | Migraine | 16/73 | 0.68 (0.39–1.18) | 0.69 (0.41–1.18) | -0.41 / -1.33 | No robust signal | Other |
| Elagolix vs Myfembree | Migraine | 16/7 | 0.29 (0.12–0.74) | 0.31 (0.13–0.74) | -0.33 / -1.26 | No robust signal | Other |
| Myfembree vs background | Dizziness | 6/106 | 1.71 (0.73–4.03) | 1.65 (0.75–3.64) | 0.63 / -1.14 | No robust signal | Other |
| Elagolix vs background | Dizziness | 15/97 | 0.47 (0.27–0.82) | 0.49 (0.29–0.83) | -0.83 / -1.72 | No robust signal | Other |
| Elagolix vs Myfembree | Dizziness | 15/6 | 0.33 (0.12–0.87) | 0.34 (0.14–0.86) | -0.29 / -1.26 | No robust signal | Other |
| Elagolix vs Myfembree | Abdominal pain | 19/2 | 1.32 (0.30–5.78) | 1.31 (0.31–5.50) | 0.04 / -0.87 | No robust signal | GI |
| Elagolix vs background | Abdominal pain | 19/80 | 0.74 (0.44–1.23) | 0.75 (0.46–1.22) | -0.32 / -1.18 | No robust signal | GI |
| Myfembree vs background | Abdominal pain | 2/97 | 0.59 (0.14–2.44) | 0.60 (0.15–2.39) | -0.60 / -2.90 | No robust signal | GI |
| Myfembree vs background | Alopecia | 3/58 | 1.53 (0.47–5.00) | 1.51 (0.48–4.71) | 0.47 / -1.86 | No robust signal | Skin/subcutaneous symptom |
| Elagolix vs background | Alopecia | 12/49 | 0.77 (0.41–1.45) | 0.77 (0.41–1.44) | -0.28 / -1.36 | No robust signal | Skin/subcutaneous symptom |
| Elagolix vs Myfembree | Alopecia | 12/3 | 0.54 (0.15–1.96) | 0.55 (0.16–1.91) | -0.13 / -1.24 | No robust signal | Skin/subcutaneous symptom |
| Elagolix vs Myfembree | Feeling abnormal | 24/0 | 7.09 (0.43–117.9) | 6.82 (0.42–111.0) | 0.16 / -0.67 | No robust signal | Other |
| Elagolix vs background | Feeling abnormal | 24/32 | 2.43 (1.42–4.16) | 2.37 (1.41–3.98) | 0.81 / -0.14 | No robust signal | Other |
| Myfembree vs background | Feeling abnormal | 0/56 | 0.25 (0.02–4.09) | 0.26 (0.02–4.09) | -1.27 / -4.63 | No robust signal | Other |
| Elagolix vs Myfembree | Adverse drug reaction | 23/0 | 6.79 (0.41–113.0) | 6.54 (0.40–106.6) | 0.15 / -0.68 | No robust signal | Other |
| Elagolix vs background | Adverse drug reaction | 23/23 | 3.25 (1.81–5.84) | 3.15 (1.78–5.58) | 1.02 / 0.01 | Robust signal | Other |
| Myfembree vs background | Adverse drug reaction | 0/46 | 0.31 (0.02–5.00) | 0.31 (0.02–4.98) | -1.05 / -4.50 | No robust signal | Other |
| Elagolix vs Myfembree | Drug ineffective | 44/1 | 6.50 (0.88–47.88) | 6.06 (0.85–43.31) | 0.15 / -0.46 | No robust signal | Disease/treatment-effect related reports |
| Elagolix vs background | Drug ineffective | 44/95 | 1.50 (1.04–2.18) | 1.46 (1.04–2.06) | 0.39 / -0.25 | No robust signal | Disease/treatment-effect related reports |
| Myfembree vs background | Drug ineffective | 1/138 | 0.20 (0.03–1.46) | 0.21 (0.03–1.49) | -1.77 / -4.39 | No robust signal | Disease/treatment-effect related reports |
| Elagolix vs Myfembree | Endometriosis | 79/3 | 4.07 (1.25–13.23) | 3.62 (1.17–11.19) | 0.13 / -0.33 | Basic signal; unstable small-cell estimate | Disease/treatment-effect related reports |
| Elagolix vs background | Endometriosis | 79/129 | 2.09 (1.55–2.82) | 1.93 (1.49–2.51) | 0.65 / 0.14 | No robust signal | Disease/treatment-effect related reports |
| Myfembree vs background | Endometriosis | 3/205 | 0.40 (0.13–1.29) | 0.43 (0.14–1.30) | -1.08 / -2.91 | No robust signal | Disease/treatment-effect related reports |

*Note.* Analyses were repeated after restricting the dataset to serious reports only. ROR, reporting odds ratio; PRR, proportional reporting ratio; IC, information component; IC025, lower 95% credibility interval of IC; PT, preferred term.

## **Table S5. Healthcare-professional-only sensitivity analysis.**

| **Comparison** | **PT** | **Cases target/comparator** | **ROR (95% CI)** | **PRR (95% CI)** | **IC / IC025** | **Signal classification** | **Clinical category** |
| --- | --- | --- | --- | --- | --- | --- | --- |
| Elagolix vs background | Hot flush | 152/70 | 3.47 (2.59–4.67) | 3.12 (2.37–4.09) | 0.73 / 0.36 | Robust signal | Vasomotor |
| Elagolix vs Myfembree | Hot flush | 152/13 | 2.70 (1.50–4.86) | 2.46 (1.42–4.25) | 0.16 / -0.18 | Basic signal | Vasomotor |
| Myfembree vs background | Hot flush | 13/209 | 0.64 (0.36–1.14) | 0.66 (0.38–1.13) | -0.54 / -1.54 | No robust signal | Vasomotor |
| Elagolix vs Myfembree | Nausea | 94/10 | 2.07 (1.06–4.04) | 1.97 (1.05–3.73) | 0.13 / -0.29 | Basic signal | GI |
| Elagolix vs background | Nausea | 94/73 | 1.93 (1.41–2.65) | 1.85 (1.38–2.48) | 0.45 / 0.00 | No robust signal | GI |
| Myfembree vs background | Nausea | 10/157 | 0.66 (0.34–1.27) | 0.67 (0.36–1.26) | -0.51 / -1.64 | No robust signal | GI |
| Elagolix vs Myfembree | Headache | 90/11 | 1.79 (0.94–3.40) | 1.72 (0.93–3.16) | 0.11 / -0.32 | No robust signal | Other |
| Elagolix vs background | Headache | 90/92 | 1.44 (1.07–1.95) | 1.40 (1.06–1.86) | 0.27 / -0.17 | No robust signal | Other |
| Myfembree vs background | Headache | 11/171 | 0.66 (0.36–1.24) | 0.68 (0.38–1.23) | -0.49 / -1.58 | No robust signal | Other |
| Elagolix vs Myfembree | Depression | 88/10 | 1.93 (0.98–3.77) | 1.85 (0.98–3.50) | 0.12 / -0.32 | No robust signal | Neuropsych/sleep |
| Elagolix vs background | Depression | 88/77 | 1.70 (1.24–2.33) | 1.64 (1.22–2.20) | 0.37 / -0.08 | No robust signal | Neuropsych/sleep |
| Myfembree vs background | Depression | 10/155 | 0.67 (0.35–1.29) | 0.68 (0.37–1.28) | -0.49 / -1.63 | No robust signal | Neuropsych/sleep |
| Elagolix vs Myfembree | Arthralgia | 77/4 | 4.28 (1.55–11.83) | 4.04 (1.50–10.93) | 0.20 / -0.27 | Basic signal; unstable small-cell estimate | Musculoskeletal |
| Elagolix vs background | Arthralgia | 77/61 | 1.88 (1.33–2.65) | 1.81 (1.31–2.51) | 0.44 / -0.05 | No robust signal | Musculoskeletal |
| Myfembree vs background | Arthralgia | 4/134 | 0.30 (0.11–0.83) | 0.32 (0.12–0.85) | -1.46 / -3.02 | No robust signal | Musculoskeletal |
| Elagolix vs Myfembree | Suicidal ideation | 66/4 | 3.63 (1.31–10.07) | 3.47 (1.28–9.41) | 0.19 / -0.32 | Basic signal; unstable small-cell estimate | Neuropsych/sleep |
| Elagolix vs background | Suicidal ideation | 66/48 | 2.04 (1.39–2.98) | 1.97 (1.37–2.84) | 0.49 / -0.05 | No robust signal | Neuropsych/sleep |
| Myfembree vs background | Suicidal ideation | 4/110 | 0.37 (0.14–1.02) | 0.39 (0.14–1.03) | -1.20 / -2.80 | No robust signal | Neuropsych/sleep |
| Elagolix vs background | Anxiety | 56/52 | 1.58 (1.07–2.32) | 1.55 (1.07–2.24) | 0.33 / -0.23 | No robust signal | Neuropsych/sleep |
| Elagolix vs Myfembree | Anxiety | 56/9 | 1.32 (0.65–2.72) | 1.31 (0.66–2.60) | 0.06 / -0.48 | No robust signal | Neuropsych/sleep |
| Myfembree vs background | Anxiety | 9/99 | 0.96 (0.48–1.93) | 0.96 (0.49–1.88) | -0.05 / -1.33 | No robust signal | Neuropsych/sleep |
| Elagolix vs Myfembree | Insomnia | 53/6 | 1.90 (0.81–4.48) | 1.86 (0.81–4.26) | 0.12 / -0.44 | No robust signal | Neuropsych/sleep |
| Elagolix vs background | Insomnia | 53/56 | 1.38 (0.94–2.02) | 1.36 (0.94–1.96) | 0.24 / -0.33 | No robust signal | Neuropsych/sleep |
| Myfembree vs background | Insomnia | 6/103 | 0.61 (0.26–1.40) | 0.62 (0.27–1.39) | -0.61 / -2.03 | No robust signal | Neuropsych/sleep |
| Myfembree vs background | Fatigue | 14/121 | 1.24 (0.70–2.20) | 1.23 (0.72–2.09) | 0.26 / -0.84 | No robust signal | Other |
| Elagolix vs background | Fatigue | 45/90 | 0.71 (0.49–1.02) | 0.72 (0.51–1.02) | -0.30 / -0.86 | No robust signal | Other |
| Elagolix vs Myfembree | Fatigue | 45/14 | 0.66 (0.36–1.23) | 0.68 (0.38–1.21) | -0.11 / -0.70 | No robust signal | Other |
| Elagolix vs background | Mood swings | 41/42 | 1.42 (0.91–2.20) | 1.40 (0.92–2.14) | 0.26 / -0.39 | No robust signal | Neuropsych/sleep |
| Elagolix vs Myfembree | Mood swings | 41/7 | 1.24 (0.55–2.80) | 1.23 (0.56–2.71) | 0.05 / -0.58 | No robust signal | Neuropsych/sleep |
| Myfembree vs background | Mood swings | 7/76 | 0.97 (0.44–2.14) | 0.98 (0.46–2.09) | -0.03 / -1.48 | No robust signal | Neuropsych/sleep |
| Elagolix vs background | Night sweats | 34/16 | 3.12 (1.71–5.68) | 3.05 (1.69–5.50) | 0.71 / -0.07 | No robust signal | Vasomotor |
| Elagolix vs Myfembree | Night sweats | 34/5 | 1.44 (0.56–3.73) | 1.43 (0.57–3.61) | 0.08 / -0.61 | No robust signal | Vasomotor |
| Myfembree vs background | Night sweats | 5/45 | 1.18 (0.46–3.01) | 1.18 (0.47–2.93) | 0.19 / -1.57 | No robust signal | Vasomotor |
| Myfembree vs background | Migraine | 8/74 | 1.15 (0.55–2.42) | 1.15 (0.56–2.34) | 0.17 / -1.25 | No robust signal | Other |
| Elagolix vs background | Migraine | 36/46 | 1.13 (0.72–1.76) | 1.12 (0.73–1.73) | 0.10 / -0.58 | No robust signal | Other |
| Elagolix vs Myfembree | Migraine | 36/8 | 0.94 (0.43–2.06) | 0.95 (0.45–2.01) | -0.01 / -0.67 | No robust signal | Other |
| Myfembree vs background | Dizziness | 7/81 | 0.91 (0.42–2.00) | 0.92 (0.43–1.96) | -0.11 / -1.54 | No robust signal | Other |
| Elagolix vs Myfembree | Dizziness | 27/7 | 0.81 (0.35–1.87) | 0.81 (0.36–1.84) | -0.06 / -0.81 | No robust signal | Other |
| Elagolix vs background | Dizziness | 27/61 | 0.63 (0.39–0.99) | 0.64 (0.41–0.99) | -0.41 / -1.13 | No robust signal | Other |
| Elagolix vs Myfembree | Abdominal pain | 23/5 | 0.97 (0.36–2.57) | 0.97 (0.37–2.51) | -0.01 / -0.83 | No robust signal | GI |
| Myfembree vs background | Abdominal pain | 5/75 | 0.70 (0.28–1.75) | 0.71 (0.29–1.73) | -0.43 / -2.02 | No robust signal | GI |
| Elagolix vs background | Abdominal pain | 23/57 | 0.57 (0.35–0.93) | 0.58 (0.36–0.93) | -0.50 / -1.27 | No robust signal | GI |
| Myfembree vs background | Alopecia | 14/64 | 2.41 (1.33–4.36) | 2.32 (1.32–4.06) | 1.00 / -0.28 | No robust signal | Skin/subcutaneous symptom |
| Elagolix vs background | Alopecia | 31/47 | 0.95 (0.60–1.50) | 0.95 (0.61–1.48) | -0.05 / -0.75 | No robust signal | Skin/subcutaneous symptom |
| Elagolix vs Myfembree | Alopecia | 31/14 | 0.45 (0.23–0.86) | 0.47 (0.25–0.86) | -0.26 / -0.94 | No robust signal | Skin/subcutaneous symptom |
| Elagolix vs Myfembree | Feeling abnormal | 31/1 | 6.68 (0.91–49.19) | 6.51 (0.89–47.45) | 0.23 / -0.52 | No robust signal | Other |
| Elagolix vs background | Feeling abnormal | 31/26 | 1.73 (1.02–2.94) | 1.71 (1.02–2.86) | 0.40 / -0.37 | No robust signal | Other |
| Myfembree vs background | Feeling abnormal | 1/56 | 0.19 (0.03–1.35) | 0.19 (0.03–1.36) | -1.85 / -4.46 | No robust signal | Other |
| Elagolix vs Myfembree | Adverse drug reaction | 49/1 | 10.75 (1.48–78.25) | 10.29 (1.43–74.15) | 0.24 / -0.35 | Basic signal; unstable small-cell estimate | Other |
| Elagolix vs background | Adverse drug reaction | 49/31 | 2.33 (1.48–3.68) | 2.27 (1.46–3.53) | 0.57 / -0.06 | No robust signal | Other |
| Myfembree vs background | Adverse drug reaction | 1/79 | 0.13 (0.02–0.94) | 0.13 (0.02–0.96) | -2.30 / -4.83 | No robust signal | Other |
| Elagolix vs Myfembree | Drug ineffective | 87/4 | 4.89 (1.78–13.47) | 4.57 (1.69–12.32) | 0.21 / -0.23 | Basic signal; unstable small-cell estimate | Disease/treatment-effect related reports |
| Elagolix vs background | Drug ineffective | 87/90 | 1.42 (1.05–1.93) | 1.39 (1.04–1.84) | 0.26 / -0.19 | No robust signal | Disease/treatment-effect related reports |
| Myfembree vs background | Drug ineffective | 4/173 | 0.23 (0.08–0.63) | 0.24 (0.09–0.65) | -1.81 / -3.32 | No robust signal | Disease/treatment-effect related reports |
| Elagolix vs Myfembree | Endometriosis | 102/6 | 3.85 (1.67–8.88) | 3.57 (1.59–8.03) | 0.19 / -0.22 | Basic signal | Disease/treatment-effect related reports |
| Elagolix vs background | Endometriosis | 102/98 | 1.55 (1.16–2.07) | 1.49 (1.15–1.95) | 0.31 / -0.11 | No robust signal | Disease/treatment-effect related reports |
| Myfembree vs background | Endometriosis | 6/194 | 0.31 (0.14–0.70) | 0.33 (0.15–0.73) | -1.45 / -2.75 | No robust signal | Disease/treatment-effect related reports |

*Note.* Analyses were repeated among healthcare-professional-submitted reports to assess the influence of reporting source. ROR, reporting odds ratio; PRR, proportional reporting ratio; IC, information component; IC025, lower 95% credibility interval of IC; PT, preferred term.

## **Table S6. Reporter-type-stratified analysis.**

| **PT** | **Consumer ROR (95% CI)** | **Consumer cases target/comparator** | **HCP ROR (95% CI)** | **HCP cases target/comparator** | **Direction consistent** | **Clinical category** |
| --- | --- | --- | --- | --- | --- | --- |
| Hot flush | 3.52 (0.83–14.97) | 106/2 | 2.70 (1.50–4.86) | 152/13 | Yes | Vasomotor |
| Nausea | 1.97 (0.46–8.46) | 65/2 | 2.07 (1.06–4.04) | 94/10 | Yes | GI |
| Headache | 2.15 (0.50–9.19) | 70/2 | 1.79 (0.94–3.40) | 90/11 | Yes | Other |
| Depression | 4.80 (0.29–79.93) | 38/0 | 1.93 (0.98–3.77) | 88/10 | Yes | Neuropsych/sleep |
| Arthralgia | 1.22 (0.28–5.27) | 42/2 | 4.28 (1.55–11.83) | 77/4 | Yes | Musculoskeletal |
| Suicidal ideation | 2.47 (0.15–41.74) | 20/0 | 3.63 (1.31–10.07) | 66/4 | Yes | Neuropsych/sleep |
| Anxiety | 0.85 (0.19–3.72) | 30/2 | 1.32 (0.65–2.72) | 56/9 | No | Neuropsych/sleep |
| Insomnia | 1.57 (0.21–11.95) | 27/1 | 1.90 (0.81–4.48) | 53/6 | Yes | Neuropsych/sleep |
| Fatigue | 1.15 (0.15–8.84) | 20/1 | 0.66 (0.36–1.23) | 45/14 | No | Other |
| Mood swings | 2.72 (0.16–45.85) | 22/0 | 1.24 (0.55–2.80) | 41/7 | Yes | Neuropsych/sleep |
| Night sweats | 3.61 (0.22–60.50) | 29/0 | 1.44 (0.56–3.73) | 34/5 | Yes | Vasomotor |
| Migraine | 0.30 (0.08–1.09) | 17/3 | 0.94 (0.43–2.06) | 36/8 | Yes | Other |
| Dizziness | 0.58 (0.13–2.61) | 21/2 | 0.81 (0.35–1.87) | 27/7 | Yes | Other |
| Abdominal pain | 1.51 (0.20–11.50) | 26/1 | 0.97 (0.36–2.57) | 23/5 | No | GI |
| Alopecia | 0.33 (0.07–1.53) | 12/2 | 0.45 (0.23–0.86) | 31/14 | Yes | Skin/subcutaneous symptom |
| Feeling abnormal | 2.34 (0.14–39.69) | 19/0 | 6.68 (0.91–49.19) | 31/1 | Yes | Other |
| Adverse drug reaction | 1.48 (0.09–25.60) | 12/0 | 10.75 (1.48–78.25) | 49/1 | Yes | Other |
| Drug ineffective | 2.72 (0.16–45.85) | 22/0 | 4.89 (1.78–13.47) | 87/4 | Yes | Disease/treatment-effect related reports |
| Endometriosis | 6.03 (0.36–100.1) | 47/0 | 3.85 (1.67–8.88) | 102/6 | Yes | Disease/treatment-effect related reports |

*Note.* Selected PT-level reporting signals were compared across consumer and healthcare-professional reports. HCP, healthcare professional; ROR, reporting odds ratio; PT, preferred term.

## **Table S7. Overlapping-market-period analysis.**

| **PT** | **Cases elagolix/Myfembree** | **ROR (95% CI)** | **PRR (95% CI)** | **IC / IC025** | **Signal classification** | **Clinical category** |
| --- | --- | --- | --- | --- | --- | --- |
| Hot flush | 90/18 | 3.97 (2.33–6.76) | 3.33 (2.05–5.39) | 0.47 / 0.01 | Robust signal | Vasomotor |
| Night sweats | 23/6 | 2.64 (1.06–6.57) | 2.55 (1.05–6.18) | 0.39 / -0.50 | Basic signal | Vasomotor |
| Nausea | 52/14 | 2.68 (1.45–4.94) | 2.47 (1.40–4.37) | 0.39 / -0.21 | Basic signal | GI |
| Abdominal pain | 9/7 | 0.85 (0.31–2.31) | 0.86 (0.32–2.27) | -0.09 / -1.37 | No robust signal | GI |
| Headache | 59/15 | 2.88 (1.60–5.20) | 2.62 (1.52–4.52) | 0.40 / -0.15 | Basic signal | Other |
| Migraine | 10/13 | 0.50 (0.22–1.16) | 0.51 (0.23–1.15) | -0.45 / -1.60 | No robust signal | Other |
| Dizziness | 14/10 | 0.93 (0.41–2.12) | 0.93 (0.42–2.07) | -0.04 / -1.08 | No robust signal | Other |
| Depression | 25/11 | 1.54 (0.75–3.19) | 1.51 (0.76–3.02) | 0.21 / -0.62 | No robust signal | Neuropsych/sleep |
| Anxiety | 22/11 | 1.35 (0.64–2.83) | 1.33 (0.66–2.70) | 0.15 / -0.72 | No robust signal | Neuropsych/sleep |
| Insomnia | 17/8 | 1.43 (0.61–3.36) | 1.41 (0.62–3.23) | 0.17 / -0.81 | No robust signal | Neuropsych/sleep |
| Mood swings | 12/7 | 1.14 (0.44–2.94) | 1.14 (0.45–2.86) | 0.07 / -1.08 | No robust signal | Neuropsych/sleep |
| Suicidal ideation | 13/7 | 1.24 (0.49–3.16) | 1.24 (0.50–3.06) | 0.11 / -1.00 | No robust signal | Neuropsych/sleep |
| Arthralgia | 33/7 | 3.32 (1.45–7.62) | 3.14 (1.41–6.99) | 0.45 / -0.30 | Basic signal | Musculoskeletal |
| Fatigue | 17/15 | 0.74 (0.36–1.51) | 0.75 (0.38–1.48) | -0.17 / -1.10 | No robust signal | Other |
| Alopecia | 6/16 | 0.24 (0.09–0.62) | 0.25 (0.10–0.63) | -1.08 / -2.42 | No robust signal | Skin/subcutaneous symptom |

*Note.* The secondary head-to-head analysis was restricted to the overlapping market period to assess potential differential market availability and time-dependent reporting bias. ROR, reporting odds ratio; PRR, proportional reporting ratio; IC, information component; IC025, lower 95% credibility interval of IC; PT, preferred term.

## **Table S8. Bootstrap validation of selected PT-level head-to-head estimates.**

| **PT** | **Original ROR (95% CI)** | **Bootstrap median ROR** | **Bootstrap 2.5–97.5 percentile** | **Signal probability** | **Stable signal** | **Interpretation** | **Clinical category** |
| --- | --- | --- | --- | --- | --- | --- | --- |
| Hot flush | 2.54 (1.57–4.12) | 2.56 | 1.65–4.54 | 1.00 | Yes | Stable direction in bootstrap analysis | Vasomotor |
| Nausea | 2.17 (1.24–3.80) | 2.20 | 1.34–4.40 | 1.00 | Yes | Stable direction in bootstrap analysis | GI |
| Headache | 1.98 (1.15–3.41) | 1.99 | 1.25–3.86 | 1.00 | Yes | Stable direction in bootstrap analysis | Other |
| Depression | 2.18 (1.17–4.09) | 2.26 | 1.30–4.99 | 1.00 | Yes | Stable direction in bootstrap analysis | Neuropsych/sleep |
| Arthralgia | 3.01 (1.39–6.52) | 3.10 | 1.58–8.75 | 1.00 | Yes | Stable direction in bootstrap analysis | Musculoskeletal |
| Suicidal ideation | 2.47 (1.14–5.37) | 2.51 | 1.25–8.29 | 1.00 | Yes | Stable direction in bootstrap analysis | Neuropsych/sleep |
| Anxiety | 1.46 (0.77–2.75) | 1.45 | 0.83–3.22 | 0.89 | Yes | Stable direction in bootstrap analysis | Neuropsych/sleep |
| Insomnia | 1.72 (0.82–3.59) | 1.79 | 0.93–4.69 | 0.95 | Yes | Stable direction in bootstrap analysis | Neuropsych/sleep |
| Fatigue | 0.76 (0.43–1.35) | 0.77 | 0.45–1.51 | 0.21 | No | Unstable or uncertain direction in bootstrap analysis | Other |
| Mood swings | 1.58 (0.72–3.48) | 1.67 | 0.81–5.44 | 0.90 | Yes | Stable direction in bootstrap analysis | Neuropsych/sleep |
| Night sweats | 1.77 (0.76–4.12) | 1.82 | 0.84–6.29 | 0.93 | Yes | Stable direction in bootstrap analysis | Vasomotor |
| Dizziness | 0.90 (0.45–1.78) | 0.91 | 0.47–1.99 | 0.39 | No | Unstable or uncertain direction in bootstrap analysis | Other |
| Alopecia | 0.45 (0.25–0.80) | 0.44 | 0.25–0.83 | 0.01 | No | Unstable or uncertain direction in bootstrap analysis | Skin/subcutaneous symptom |

*Note.* Bootstrap resampling was performed at the report level to assess directional stability of selected head-to-head ROR estimates. Results were used as supportive robustness information rather than evidence of causality. ROR, reporting odds ratio; PT, preferred term.

## **Table S9. Association of selected PTs with serious reporting.**

| **Drug group** | **PT** | **PT reports, n** | **Serious reports, n (%)** | **Non-serious reports, n** | **OR for serious reporting (95% CI)** |
| --- | --- | --- | --- | --- | --- |
| Elagolix | Suicidal ideation | 104 | 100 (96.2%) | 4 | 67.13 (24.57–183.5) |
| Elagolix | Depression | 143 | 58 (40.6%) | 85 | 1.56 (1.10–2.22) |
| Elagolix | Anxiety | 98 | 32 (32.7%) | 66 | 1.07 (0.69–1.65) |
| Elagolix | Nausea | 179 | 50 (27.9%) | 129 | 0.84 (0.59–1.18) |
| Elagolix | Insomnia | 84 | 19 (22.6%) | 65 | 0.63 (0.37–1.06) |
| Elagolix | Night sweats | 65 | 15 (23.1%) | 50 | 0.65 (0.36–1.17) |
| Elagolix | Hot flush | 272 | 60 (22.1%) | 212 | 0.58 (0.42–0.78) |
| Myfembree | Alopecia | 16 | 3 (18.8%) | 13 | 0.62 (0.17–2.22) |
| Myfembree | Dysmenorrhoea | 10 | 1 (10.0%) | 9 | 0.29 (0.04–2.36) |
| Myfembree | Heavy menstrual bleeding | 17 | 2 (11.8%) | 15 | 0.35 (0.08–1.56) |
| Myfembree | Intermenstrual bleeding | 18 | 0 (0.0%) | 18 | 0.07 (0.00–1.13) |
| Myfembree | Menstruation irregular | 9 | 0 (0.0%) | 9 | 0.14 (0.01–2.38) |
| Myfembree | Breast tenderness | 4 | 0 (0.0%) | 4 | 0.30 (0.02–5.57) |

*Note.* Odds ratios evaluate the association between each selected PT and serious reporting status within each drug group. Estimates based on small numbers of reports should be interpreted cautiously. OR, odds ratio; PT, preferred term.

## **Table S10. Counts and serious-reporting proportions for selected PTs.**

| **Drug group** | **PT** | **Reports, n** | **Serious, n** | **Non-serious, n** | **Serious reports (%)** |
| --- | --- | --- | --- | --- | --- |
| Elagolix | Hot flush | 272 | 60 | 212 | 22.1% |
| Elagolix | Nausea | 179 | 50 | 129 | 27.9% |
| Elagolix | Depression | 143 | 58 | 85 | 40.6% |
| Elagolix | Suicidal ideation | 104 | 100 | 4 | 96.2% |
| Elagolix | Anxiety | 98 | 32 | 66 | 32.7% |
| Elagolix | Insomnia | 84 | 19 | 65 | 22.6% |
| Elagolix | Night sweats | 65 | 15 | 50 | 23.1% |
| Elagolix | Dysmenorrhoea | 46 | 12 | 34 | 26.1% |
| Elagolix | Alopecia | 46 | 12 | 34 | 26.1% |
| Elagolix | Heavy menstrual bleeding | 27 | 12 | 15 | 44.4% |
| Elagolix | Intermenstrual bleeding | 16 | 4 | 12 | 25.0% |
| Elagolix | Menstruation irregular | 13 | 2 | 11 | 15.4% |
| Elagolix | Breast tenderness | 3 | 0 | 3 | 0.0% |
| Myfembree | Hot flush | 19 | 7 | 12 | 36.8% |
| Myfembree | Nausea | 14 | 5 | 9 | 35.7% |
| Myfembree | Depression | 11 | 3 | 8 | 27.3% |
| Myfembree | Suicidal ideation | 7 | 7 | 0 | 100.0% |
| Myfembree | Anxiety | 11 | 3 | 8 | 27.3% |
| Myfembree | Insomnia | 8 | 3 | 5 | 37.5% |
| Myfembree | Night sweats | 6 | 4 | 2 | 66.7% |
| Myfembree | Dysmenorrhoea | 10 | 1 | 9 | 10.0% |
| Myfembree | Alopecia | 16 | 3 | 13 | 18.8% |
| Myfembree | Heavy menstrual bleeding | 17 | 2 | 15 | 11.8% |
| Myfembree | Intermenstrual bleeding | 18 | 0 | 18 | 0.0% |
| Myfembree | Menstruation irregular | 9 | 0 | 9 | 0.0% |
| Myfembree | Breast tenderness | 4 | 0 | 4 | 0.0% |

*Note.* This table summarizes the number of selected PT reports and the proportion classified as serious within each drug group. Percentages based on small cell counts should be interpreted cautiously. PT, preferred term.

## **Table S11. Annual reporting trends.**

| **Year** | **Drug group** | **Total reports** | **Serious reports** | **Selected PT reports** | **Hot flush** | **Nausea** | **Depression** | **Headache** | **Night sweats** | **Suicidal ideation** | **Alopecia** | **Arthralgia** |
| --- | --- | --- | --- | --- | --- | --- | --- | --- | --- | --- | --- | --- |
| 2015 | Elagolix | 0 | 0 | 0 | 0 | 0 | 0 | 0 | 0 | 0 | 0 | 0 |
| 2015 | Myfembree | 0 | 0 | 0 | 0 | 0 | 0 | 0 | 0 | 0 | 0 | 0 |
| 2016 | Elagolix | 0 | 0 | 0 | 0 | 0 | 0 | 0 | 0 | 0 | 0 | 0 |
| 2016 | Myfembree | 0 | 0 | 0 | 0 | 0 | 0 | 0 | 0 | 0 | 0 | 0 |
| 2017 | Elagolix | 0 | 0 | 0 | 0 | 0 | 0 | 0 | 0 | 0 | 0 | 0 |
| 2017 | Myfembree | 0 | 0 | 0 | 0 | 0 | 0 | 0 | 0 | 0 | 0 | 0 |
| 2018 | Elagolix | 32 | 9 | 11 | 3 | 8 | 1 | 1 | 1 | 1 | 0 | 0 |
| 2018 | Myfembree | 0 | 0 | 0 | 0 | 0 | 0 | 0 | 0 | 0 | 0 | 0 |
| 2019 | Elagolix | 530 | 178 | 235 | 55 | 53 | 53 | 49 | 12 | 47 | 18 | 35 |
| 2019 | Myfembree | 0 | 0 | 0 | 0 | 0 | 0 | 0 | 0 | 0 | 0 | 0 |
| 2020 | Elagolix | 409 | 122 | 162 | 69 | 31 | 38 | 31 | 10 | 29 | 12 | 27 |
| 2020 | Myfembree | 0 | 0 | 0 | 0 | 0 | 0 | 0 | 0 | 0 | 0 | 0 |
| 2021 | Elagolix | 224 | 82 | 89 | 31 | 25 | 20 | 26 | 12 | 9 | 7 | 22 |
| 2021 | Myfembree | 0 | 0 | 0 | 0 | 0 | 0 | 0 | 0 | 0 | 0 | 0 |
| 2022 | Elagolix | 353 | 65 | 174 | 80 | 41 | 23 | 48 | 18 | 8 | 6 | 27 |
| 2022 | Myfembree | 8 | 1 | 2 | 1 | 1 | 0 | 1 | 0 | 0 | 0 | 0 |
| 2023 | Elagolix | 94 | 32 | 45 | 22 | 15 | 3 | 15 | 7 | 7 | 1 | 10 |
| 2023 | Myfembree | 92 | 13 | 24 | 5 | 5 | 4 | 4 | 2 | 2 | 7 | 2 |
| 2024 | Elagolix | 59 | 30 | 18 | 11 | 5 | 3 | 4 | 4 | 2 | 1 | 3 |
| 2024 | Myfembree | 98 | 10 | 25 | 4 | 4 | 5 | 6 | 1 | 1 | 5 | 2 |
| 2025 | Elagolix | 42 | 27 | 6 | 1 | 1 | 2 | 2 | 1 | 1 | 1 | 1 |
| 2025 | Myfembree | 54 | 33 | 13 | 5 | 2 | 1 | 2 | 2 | 1 | 4 | 2 |
| 2026 | Elagolix | 1 | 0 | 0 | 0 | 0 | 0 | 0 | 0 | 0 | 0 | 0 |
| 2026 | Myfembree | 28 | 18 | 12 | 4 | 2 | 1 | 2 | 1 | 3 | 0 | 1 |

*Note.* Annual total reports, serious reports, and selected PT reports are summarized by drug group. Data for 2026 include Q1 only and should not be interpreted as full-year trends. PT, preferred term.

## **Table S14. Top 20 PT-level reporting signals by drug.**

| **Drug group** | **PT** | **Target cases, n** | **Comparator cases, n** | **ROR (95% CI)** | **PRR (95% CI)** | **IC / IC025** | **Signal classification** |
| --- | --- | --- | --- | --- | --- | --- | --- |
| Elagolix | Night sweats | 65 | 32 | 3.21 (2.09–4.92) | 3.13 (2.06–4.75) | 0.76 / 0.19 | Strict signal |
| Elagolix | Hot flush | 272 | 151 | 3.10 (2.52–3.82) | 2.77 (2.29–3.35) | 0.71 / 0.43 | Strict signal |
| Elagolix | Maternal exposure during pregnancy | 51 | 26 | 3.08 (1.91–4.96) | 3.02 (1.89–4.82) | 0.74 / 0.10 | Strict signal |
| Elagolix | Suicidal ideation | 104 | 78 | 2.12 (1.57–2.86) | 2.05 (1.54–2.74) | 0.53 / 0.10 | Strict signal |
| Elagolix | Initial insomnia | 6 | 0 | 20.07 (1.13–356.6) | 20.00 (1.13–354.9) | 1.10 / -0.79 | Basic signal; small-cell/unstable |
| Elagolix | Placenta praevia | 6 | 0 | 20.07 (1.13–356.6) | 20.00 (1.13–354.9) | 1.10 / -0.79 | Basic signal; small-cell/unstable |
| Elagolix | Liver function test increased | 7 | 1 | 10.81 (1.33–87.96) | 10.77 (1.33–87.49) | 1.04 / -0.77 | Basic signal; small-cell/unstable |
| Elagolix | Intentional self-injury | 6 | 1 | 9.26 (1.11–77.00) | 9.23 (1.11–76.64) | 1.00 / -0.92 | Basic signal; small-cell/unstable |
| Elagolix | Alanine aminotransferase increased | 12 | 4 | 4.64 (1.49–14.42) | 4.62 (1.49–14.29) | 0.88 / -0.47 | Basic signal; small-cell/unstable |
| Elagolix | Menstruation delayed | 12 | 4 | 4.64 (1.49–14.42) | 4.62 (1.49–14.29) | 0.88 / -0.47 | Basic signal; small-cell/unstable |
| Elagolix | Hepatic enzyme increased | 19 | 7 | 4.21 (1.77–10.04) | 4.18 (1.76–9.92) | 0.86 / -0.21 | Basic signal |
| Elagolix | Abnormal uterine bleeding | 15 | 7 | 3.32 (1.35–8.15) | 3.30 (1.35–8.07) | 0.76 / -0.42 | Basic signal |
| Elagolix | Mood altered | 34 | 19 | 2.79 (1.59–4.91) | 2.75 (1.58–4.81) | 0.69 / -0.09 | Basic signal |
| Elagolix | Live birth | 17 | 10 | 2.63 (1.20–5.76) | 2.62 (1.20–5.70) | 0.65 / -0.43 | Basic signal |
| Elagolix | Contusion | 16 | 10 | 2.48 (1.12–5.47) | 2.46 (1.12–5.41) | 0.62 / -0.49 | Basic signal |
| Elagolix | Crying | 31 | 20 | 2.41 (1.37–4.24) | 2.39 (1.36–4.17) | 0.61 / -0.19 | Basic signal |
| Elagolix | Menopause | 14 | 9 | 2.41 (1.04–5.57) | 2.39 (1.04–5.52) | 0.60 / -0.58 | Basic signal |
| Elagolix | Unevaluable event | 23 | 16 | 2.23 (1.17–4.23) | 2.21 (1.17–4.18) | 0.57 / -0.35 | Basic signal |
| Elagolix | Emotional disorder | 23 | 18 | 1.98 (1.07–3.68) | 1.97 (1.06–3.63) | 0.50 / -0.41 | Basic signal |
| Elagolix | Adverse drug reaction | 63 | 52 | 1.90 (1.31–2.75) | 1.86 (1.30–2.68) | 0.47 / -0.08 | Basic signal |
| Myfembree | Therapy interrupted | 76 | 3 | 514.7 (161.0–1645.7) | 375.3 (119.1–1182.1) | 3.80 / 2.55 | Strict signal; small-cell/unstable |
| Myfembree | Intermenstrual bleeding | 18 | 38 | 7.43 (4.18–13.20) | 7.02 (4.06–12.13) | 2.19 / 0.64 | Strict signal |
| Myfembree | Heavy menstrual bleeding | 17 | 52 | 5.09 (2.90–8.93) | 4.84 (2.84–8.26) | 1.85 / 0.40 | Strict signal |
| Myfembree | Haematochezia | 6 | 5 | 18.14 (5.50–59.83) | 17.78 (5.46–57.89) | 2.44 / -0.37 | Basic signal |
| Myfembree | Insurance issue | 5 | 5 | 15.07 (4.34–52.35) | 14.81 (4.31–50.87) | 2.28 / -0.64 | Basic signal |
| Myfembree | Breast tenderness | 4 | 8 | 7.50 (2.24–25.06) | 7.41 (2.24–24.45) | 1.84 / -1.01 | Basic signal; small-cell/unstable |
| Myfembree | Inappropriate schedule of product administration | 7 | 20 | 5.29 (2.22–12.62) | 5.18 (2.21–12.16) | 1.76 / -0.40 | Basic signal |
| Myfembree | Blood oestrogen decreased | 3 | 9 | 4.98 (1.34–18.50) | 4.94 (1.34–18.14) | 1.48 / -1.46 | Basic signal; small-cell/unstable |
| Myfembree | Brain fog | 4 | 13 | 4.61 (1.49–14.23) | 4.56 (1.50–13.89) | 1.51 / -1.10 | Basic signal; small-cell/unstable |
| Myfembree | Nightmare | 3 | 10 | 4.48 (1.23–16.38) | 4.44 (1.23–16.06) | 1.40 / -1.48 | Basic signal; small-cell/unstable |
| Myfembree | Menstruation irregular | 9 | 33 | 4.14 (1.96–8.74) | 4.04 (1.95–8.36) | 1.59 / -0.25 | Basic signal |
| Myfembree | Alopecia | 16 | 108 | 2.27 (1.32–3.89) | 2.19 (1.32–3.66) | 0.98 / -0.22 | Basic signal |
| Myfembree | Dysmenorrhoea | 10 | 74 | 2.04 (1.04–3.99) | 2.00 (1.05–3.83) | 0.85 / -0.61 | Basic signal |
| Myfembree | Drug effect less than expected | 2 | 1 | 29.83 (2.70–330.0) | 29.63 (2.69–325.8) | 1.86 / -1.99 | No signal; small-cell/unstable |
| Myfembree | Nipple pain | 2 | 1 | 29.83 (2.70–330.0) | 29.63 (2.69–325.8) | 1.86 / -1.99 | No signal; small-cell/unstable |
| Myfembree | Ischaemic stroke | 2 | 2 | 14.91 (2.09–106.3) | 14.81 (2.09–104.8) | 1.73 / -1.99 | No signal; small-cell/unstable |
| Myfembree | Abdominal tenderness | 1 | 2 | 7.43 (0.67–82.20) | 7.41 (0.67–81.44) | 1.12 / -2.99 | No signal; small-cell/unstable |
| Myfembree | Angiopathy | 1 | 2 | 7.43 (0.67–82.20) | 7.41 (0.67–81.44) | 1.12 / -2.99 | No signal; small-cell/unstable |
| Myfembree | Anovulatory cycle | 1 | 2 | 7.43 (0.67–82.20) | 7.41 (0.67–81.44) | 1.12 / -2.99 | No signal; small-cell/unstable |
| Myfembree | Blood loss anaemia | 1 | 2 | 7.43 (0.67–82.20) | 7.41 (0.67–81.44) | 1.12 / -2.99 | No signal; small-cell/unstable |

*Note.* Top PT-level reporting signals are shown for elagolix and Myfembree drug-specific case–noncase analyses. ROR, reporting odds ratio; PRR, proportional reporting ratio; IC, information component; IC025, lower 95% credibility interval of IC; PT, preferred term.
